# 173. Oral Tebipenem Pivoxil Hydrobromide versus Intravenous Imipenem-Cilastatin in Patients with Complicated Urinary Tract Infections or Acute Pyelonephritis: Efficacy and Safety Results from the Phase 3 PIVOT-PO study

**DOI:** 10.1093/ofid/ofaf695.003

**Published:** 2026-01-11

**Authors:** David K Hong, Sibel Ascioglu, Nivedita Bhatt, Ian A Critchley, Masha Gaber, Leanne B Gasink, Kamal A Hamed, Aubri Hutchins, Aoibhinn McDonnell, Tal Otiker, Yasmin Sánchez-Pearson, Amanda J Sheets, Dan Sotolongo, Mike Sprys, Didem Torumkuney, Kamil Wrzosek, Amanda Peppercorn

**Affiliations:** Spero Therapeutics Inc., Cambridge, MA, USA, Cambridge, Massachusetts; GSK, London, UK, London, England, United Kingdom; Spero Therapeutics, Cambridge, Massachusetts; Spero Therapeutics Inc., Cambridge, MA, USA, Cambridge, Massachusetts; Spero Therapeutics Inc., Cambridge, MA, USA, Cambridge, Massachusetts; Spero Therapeutics Inc., Cambridge, MA, USA, Cambridge, Massachusetts; Spero Therapeutics Inc., Cambridge, MA, USA, Cambridge, Massachusetts; Spero Therapeutics Inc., Cambridge, MA, USA, Cambridge, Massachusetts; GSK, Stevenage, UK, Stevenage, England, United Kingdom; GSK, London, UK, London, England, United Kingdom; GSK, London, UK, London, England, United Kingdom; GSK, Collegeville, PA, USA, Collegeville, Pennsylvania; Links Clinical Trials, LLC, Miami, FL, USA, Miami, Florida; GSK, Philadelphia, PA, USA, Philadelphia, Pennsylvania; GSK, Brentford, England, United Kingdom; Czerniakowski Hospital, Warsaw, Poland, Warsaw, Mazowieckie, Poland; GlaxoSmithKline, Cambridge, Massachusetts

## Abstract

**Background:**

Management of complicated urinary tract infections (cUTIs) is increasingly difficult due to rising antimicrobial resistance. There is an unmet need for oral treatment options for antimicrobial-resistant cUTIs. Tebipenem pivoxil hydrobromide (TBP-PI-HBr) is an investigational oral carbapenem with activity against antimicrobial-resistant Enterobacterales, including extended-spectrum β-lactamase–positive (ESBL+) pathogens.

**Figure:**
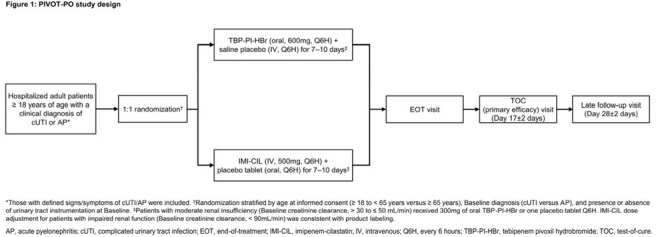

**Figure:**
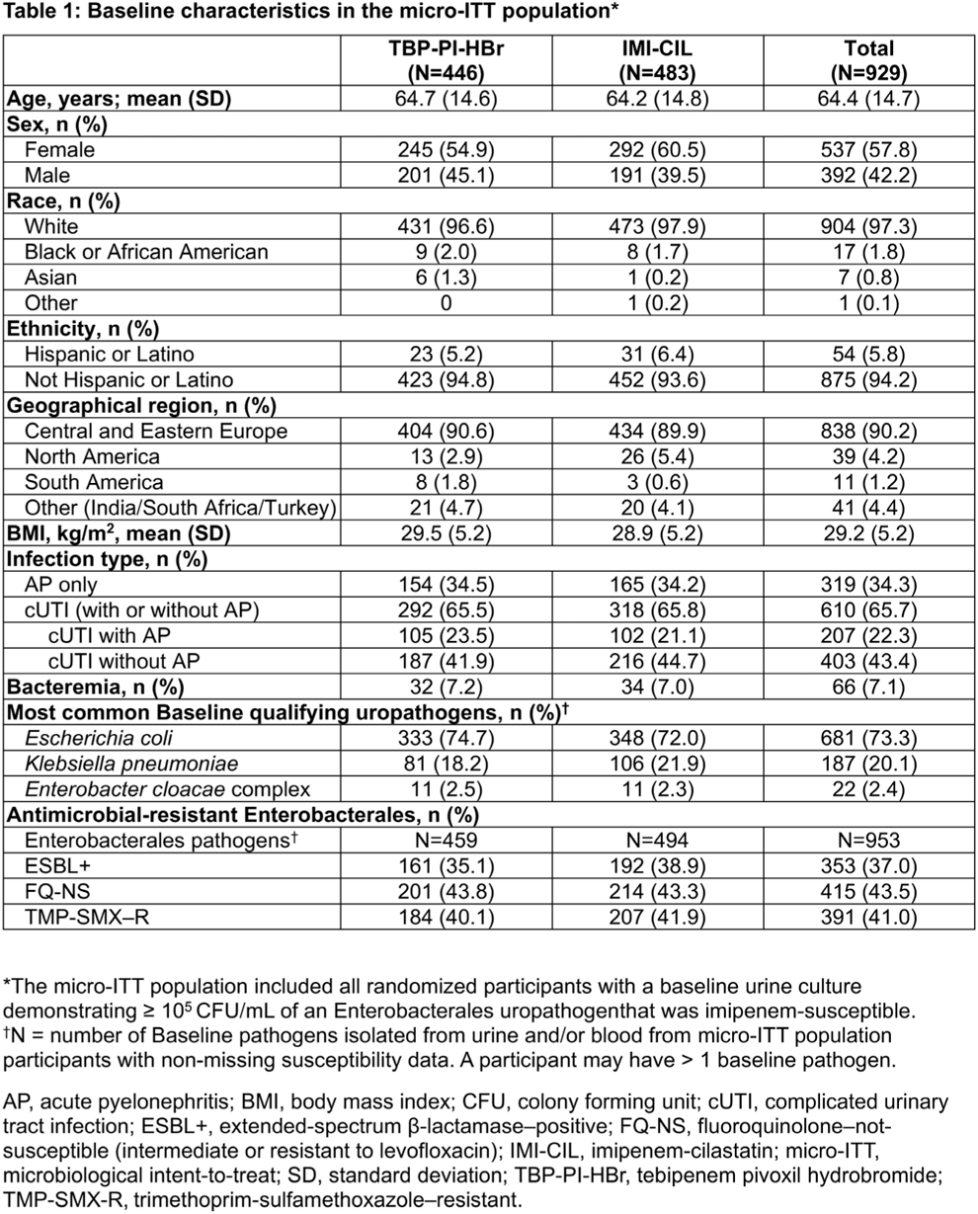

**Methods:**

PIVOT-PO (NCT06059846) was a global, randomized, double-blind, non-inferiority (10% margin) Phase 3 study comparing the efficacy and safety of oral TBP-PI-HBr with intravenous (IV) imipenem-cilastatin (IMI-CIL) in hospitalized adult patients with cUTI or acute pyelonephritis (AP) (Figure 1). The primary endpoint of overall response (clinical cure plus microbiological eradication) at test-of-cure (TOC) was assessed in the microbiological intent-to-treat (micro-ITT) population. An interim analysis to assess efficacy and futility was reviewed by an Independent Data Monitoring Committee and the study was stopped for efficacy.

**Figure:**
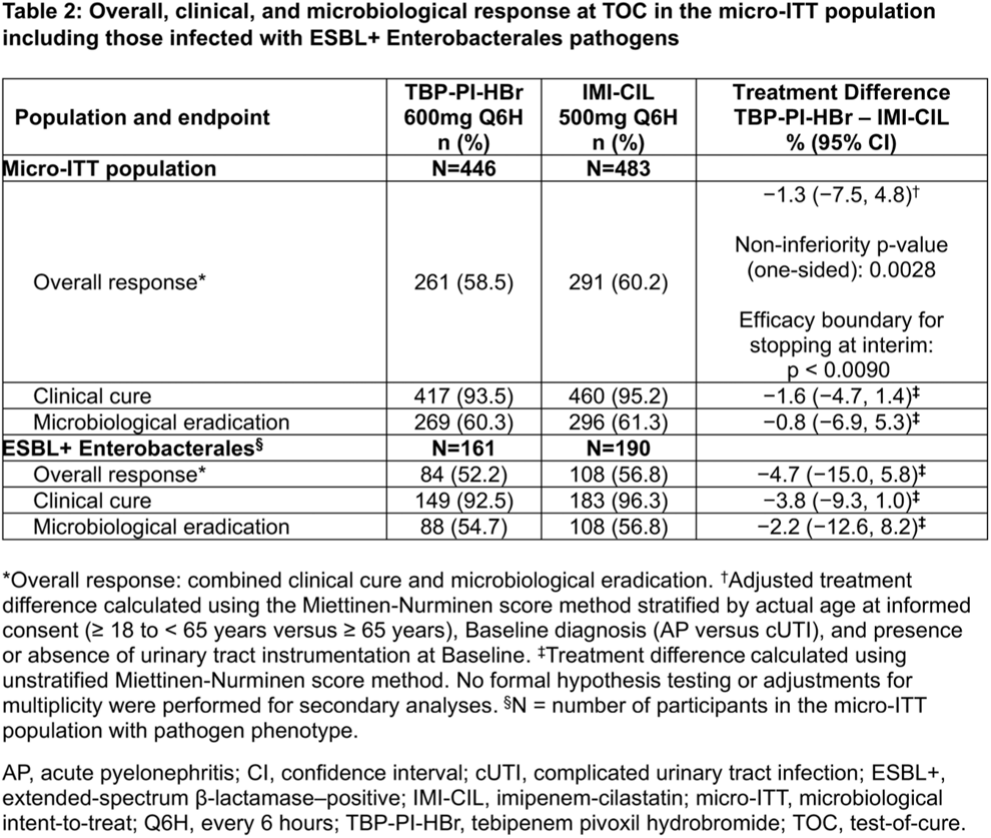

**Figure:**
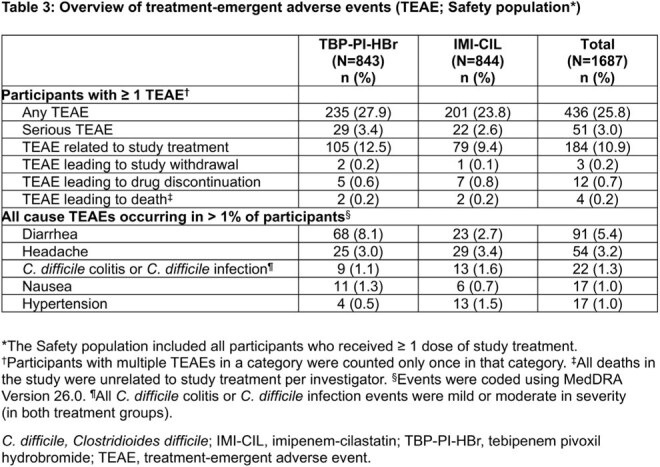

**Results:**

Baseline characteristics were balanced between treatment groups (Table 1). Overall response at TOC was 58.5% in 261/446 participants who received TBP-PI-HBr versus 60.2% in 291/483 participants who received IMI-CIL (adjusted treatment difference: −1.3%; 95% CI: −7.5%, 4.8%). Treatment effects were comparable in participants with ESBL+ Enterobacterales (Table 2). The safety profile of TBP-PI-HBr was overall consistent with IMI-CIL; the two most frequent treatment-emergent adverse events were diarrhea and headache (Table 3).

**Conclusion:**

Oral TBP-PI-HBr was non-inferior to IV IMI-CIL in the treatment of cUTI or AP and showed comparable efficacy in participants with ESBL+ Enterobacterales. No new safety signals were identified. TBP-PI-HBr may provide an effective oral treatment option for cUTI or AP.

**Funding:** PIVOT-PO was funded by Spero Therapeutics, Inc. The development of TBP-PI-HBr is supported in part with federal funds from the U.S. Department of Health and Human Services; Administration for Strategic Preparedness and Response; Biomedical Advanced Research and Development Authority (BARDA), under contract number HHSO100201800015C.

**Disclosures:**

All Authors: No reported disclosures

